# THE POTENTIAL OF MULTIDOMAIN MOBILE PROGRAMS IN SUPPORTING COGNITIVE HEALTH OF SENIORS IN RESIDENTIAL FACILITIES

**DOI:** 10.1093/geroni/igad104.3651

**Published:** 2023-12-21

**Authors:** Junhyoung Kim, Yongseop Kim, Jeff Cho, Myungjin Ko, Marcia Ory

**Affiliations:** Texas A&M University, College Station, Texas, United States; Indiana University, Bloomington, Indiana, United States; Korea International School Jeju, Seogwipo, Jeju, Republic of Korea; Silvia Health, Seoul, Republic of Korea; Texas A&M University, College Station, Texas, United States

## Abstract

This study pilot tested the efficacy of a mobile-based multidomain application, Silvia Program, on cognitive functioning of residents in assisted living facilities (ALFs). After a 12-week randomized pilot trial using a two-group pretest-posttest design (Silvia group vs. usual care group), we assessed the cognitive functioning using the Montreal Cognitive Assessment (MoCA). Results are that the total MoCA scores of the Silvia group showed significant improvement while the total scores of the control group declined. The present study provides suggestive evidence for an overall positive effect of the use of the Silvia Program on the cognitive functioning among residents in ALFs.

